# Supplementary material for: Bone mineral loss damages renal tubules in mice
Source: Commun Biol. 2026 Jan 22;9:304. doi: 10.1038/s42003-026-09603-0 (PMC12929778; doi:10.1038/s42003-026-09603-0)
Supplement: Supplementary file 2 — Supplementary Information [file 42003_2026_9603_MOESM2_ESM.pdf]

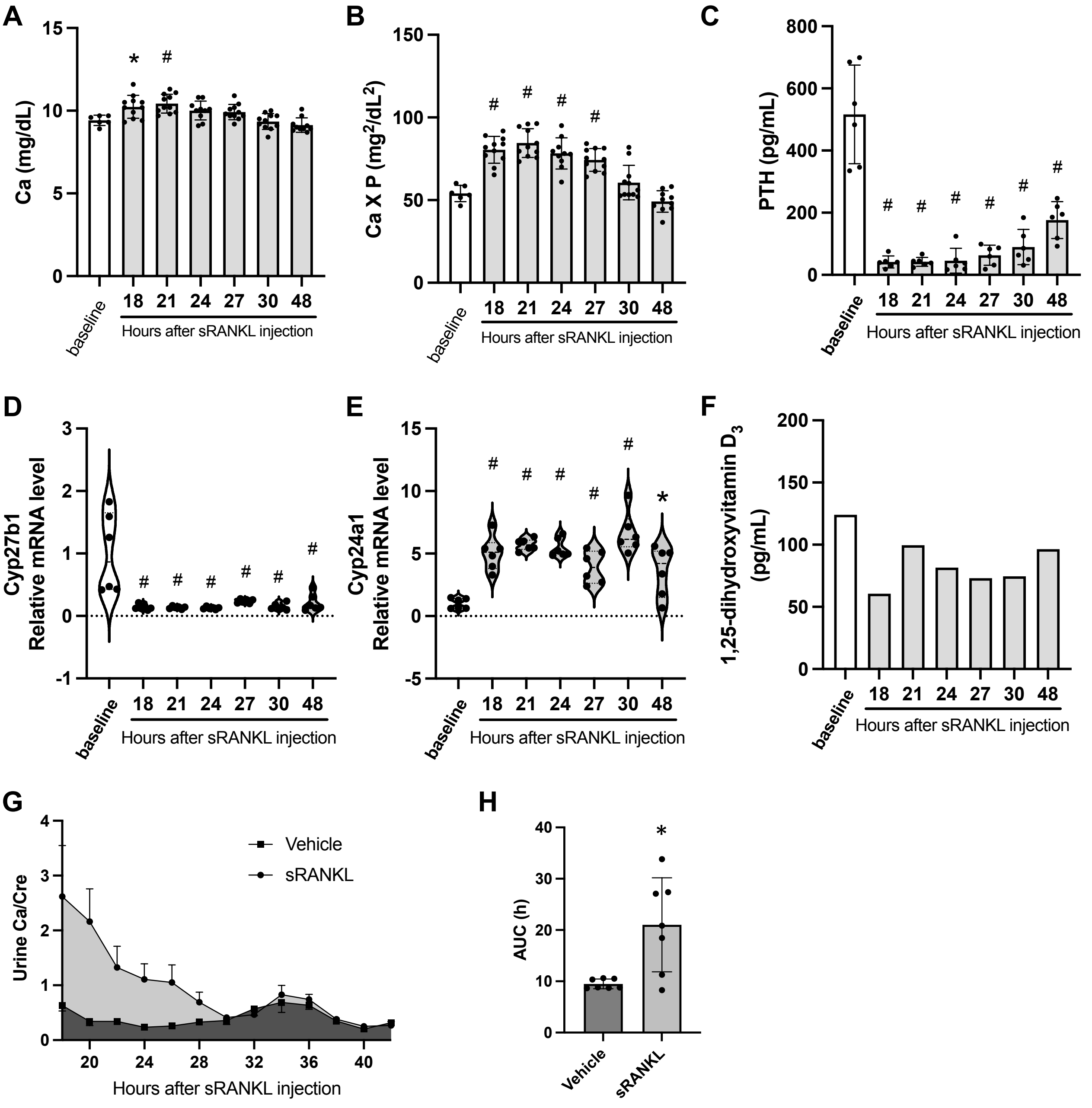

Supplementary Figure S1 | Effects of the single dose sRANKL on calcium metabolism. Mice were sacrificed before (baseline) or after intraperitoneal administration with sRANKL (2 mg/kg) at the indicated time points to harvest kidney and blood. Plasma levels of calcium (**A**), calcium phosphate product (**B**), and parathyroid hormone (PTH) (**C**). Data are presented as means ± SD. $N = 6$-12 for each column. *$P < 0.05$, #$P < 0.01$ versus baseline by one-way ANOVA with Tukey's multiple comparison test. Relative mRNA levels of *Cyp27b1* (**D**) and *Cyp24a1* (**E**) in the kidney were measured by quantitative RT-PCR. Data are indicated as violin plots with the median and quartiles (dotted lines). $N = 6$ for each column. *$P < 0.05$, #$P < 0.01$ versus baseline by one-way ANOVA with Tukey's multiple comparison test. (**F**) Plasma levels of active vitamin D (1,25-dihydroxyvitamin D_3). As the measurement of active vitamin D requires a large volume of plasma, equal amounts of plasma samples from individual mice in each time points were mixed and then subjected to radioimmunoassay for 1,25-dihydroxyvitamin D_3. Statistical analysis was not performed. (**G**) Urine calcium/creatinine ratios (Urine Ca/Cre). Spot urine samples were harvested after intraperitoneal injection of sRANKL (solid circles) or vehicle (solid squares) every 2 hours at the indicated time points. Data are presented as means ± SEM. $N = 3$-7 for each time point. (**H**) Area under the curve (AUC) of the urine calcium/creatinine ratios in individual mice injected with vehicle or sRANKL. Data are presented as means ± SD. $N = 7$ for each column. *$P < 0.05$ versus vehicle by Welch's t test.

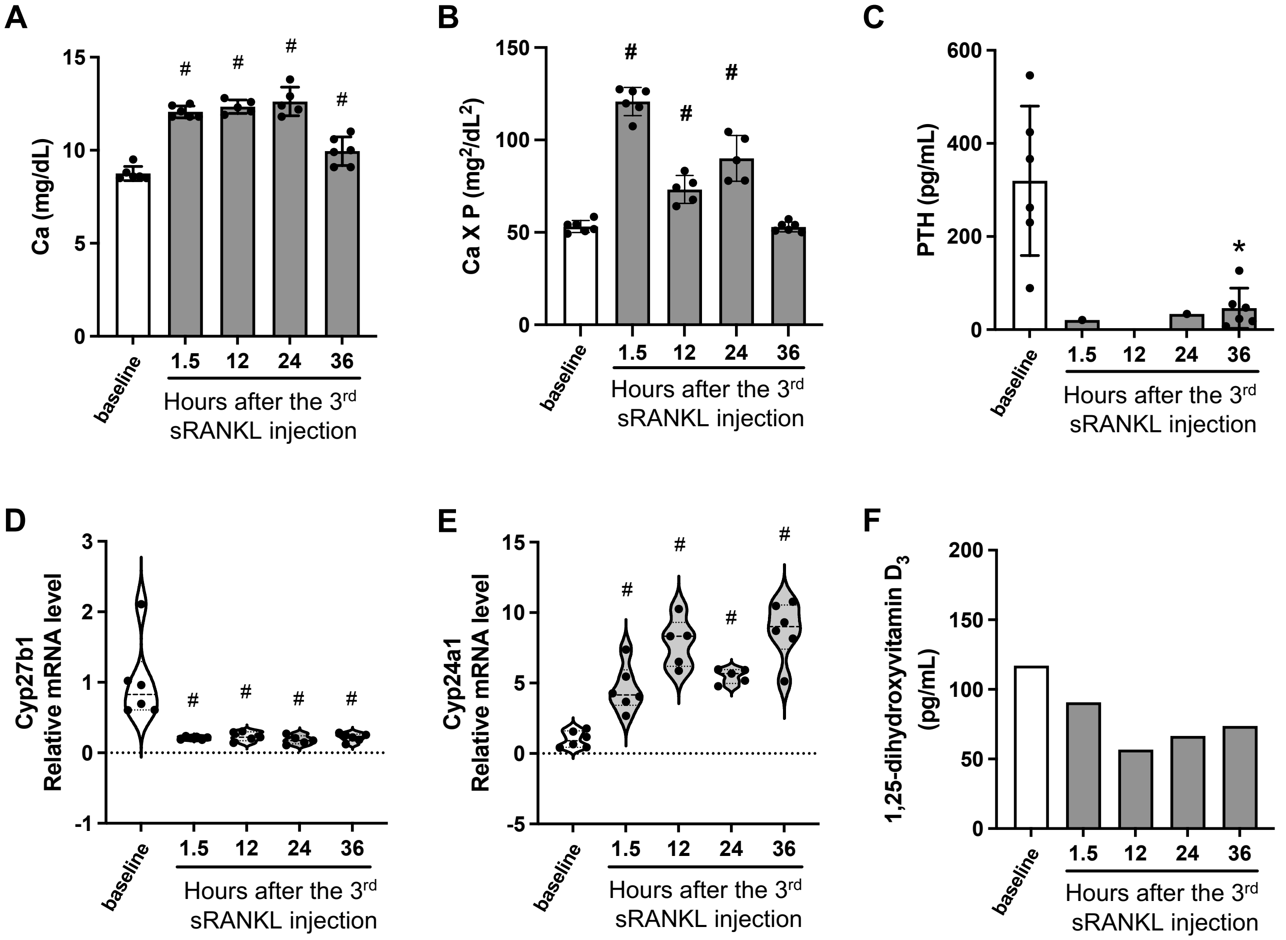

Supplementary Figure S2 | Effects of the triple dose sRANKL on calcium metabolism. Mice were sacrificed before (baseline) or after intraperitoneal administration with sRANKL (2 mg/kg, every 24 hours for 3 times) at the indicated time points to harvest kidney, femur, and blood. The femurs were used in **Figure 2**. Plasma levels of calcium (**A**), calcium phosphate product (**B**), and parathyroid hormone (PTH) (**C**). Data are presented as means ± SD. $N = 6$-12 for each column. $^{*}P < 0.05$, $^{\#}P < 0.01$ versus baseline by one-way ANOVA with Tukey's multiple comparison test. Relative mRNA levels of *Cyp27b1* (**D**) and *Cyp24a1* (**E**) in the kidney were measured by quantitative RT-PCR. Data are indicated as violin plots with the median and quartiles (dotted lines). $N = 6$ for each column. $^{\#}P < 0.01$ versus baseline by Kruskal–Wallis's test with Dunn's multiple-comparison test. (**F**) Plasma levels of active vitamin D (1,25-dihydroxyvitamin D_3) in mixed plasma samples in each time points as in **Supplementary Figure S1F**. Statistical analysis was not performed.

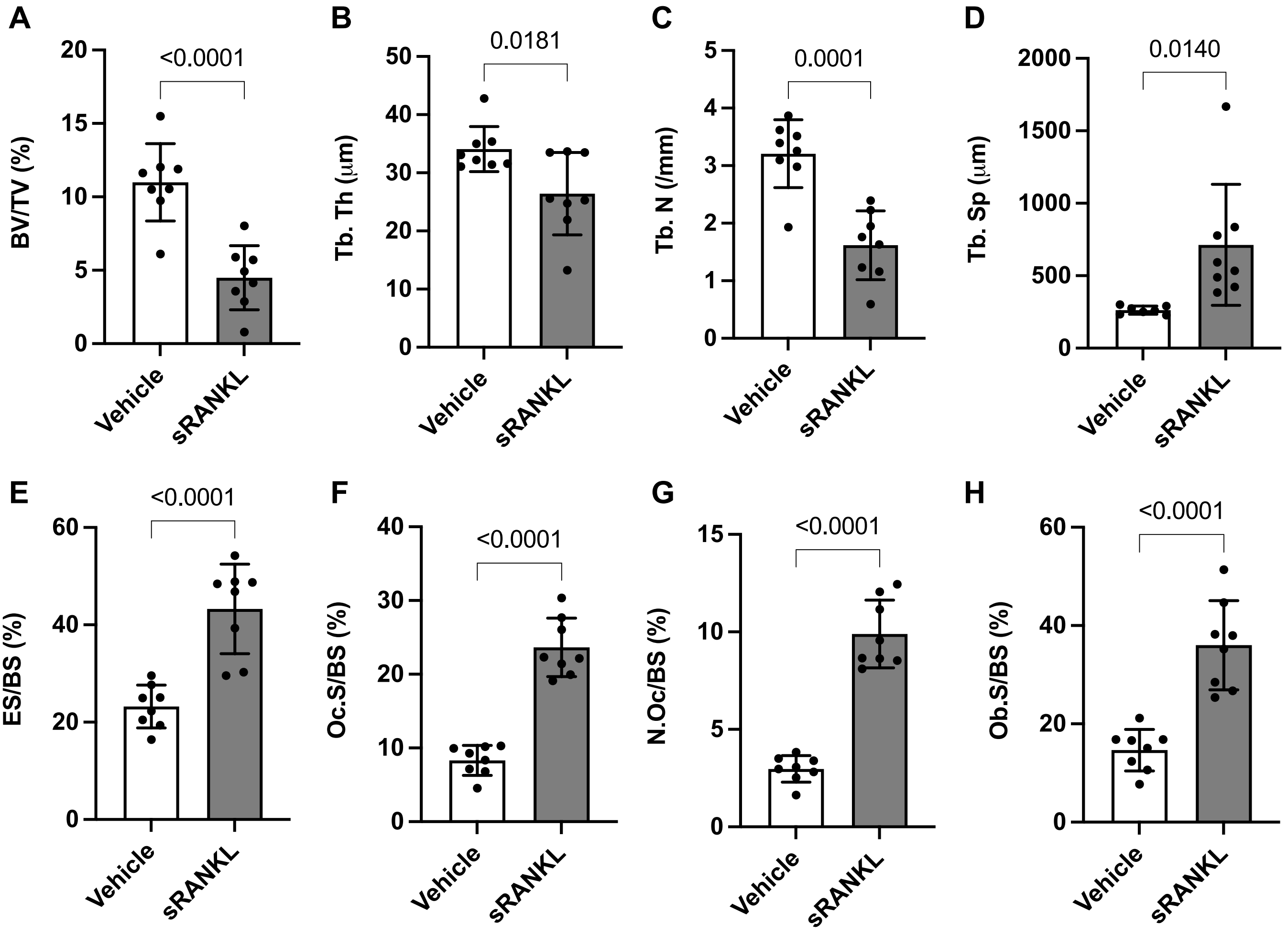

Supplementary Figure S3 | Histomorphometric analysis of the femurs from mice treated with the triple dose regimen of sRANKL. Mice were sacrificed 36 hours after the last sRANKL or vehicle injection. Trabecular bone volume fraction (**A**), trabecular thickness (**B**), trabecular number (**C**), trabecular separation (**D**), eroded surface over bone surface (**E**), osteoclast surface over bone surface (**F**), osteoclast number over bone surface (**G**), and osteoblast surface over bone surface (**H**) were shown. Data are presented as means ± SD. $N = 8$ for each column. P values by t-test are indicated.

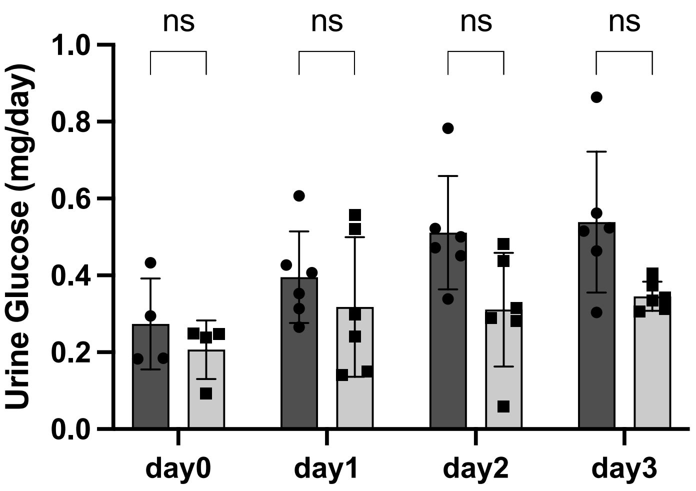

Supplementary Figure S4 | Effects of the triple dose sRANKL on urinary glucose excretion. Daily urinary glucose excretion in mice administered vehicle (black bars, solid circles) or sRANKL (gray bars, solid squares) as described in **Figure 2**. Data are presented as means ± SD. N = 4~6 per group. No significant differences were observed between the vehicle and sRANKL groups, as determined by mixed-effects analysis.

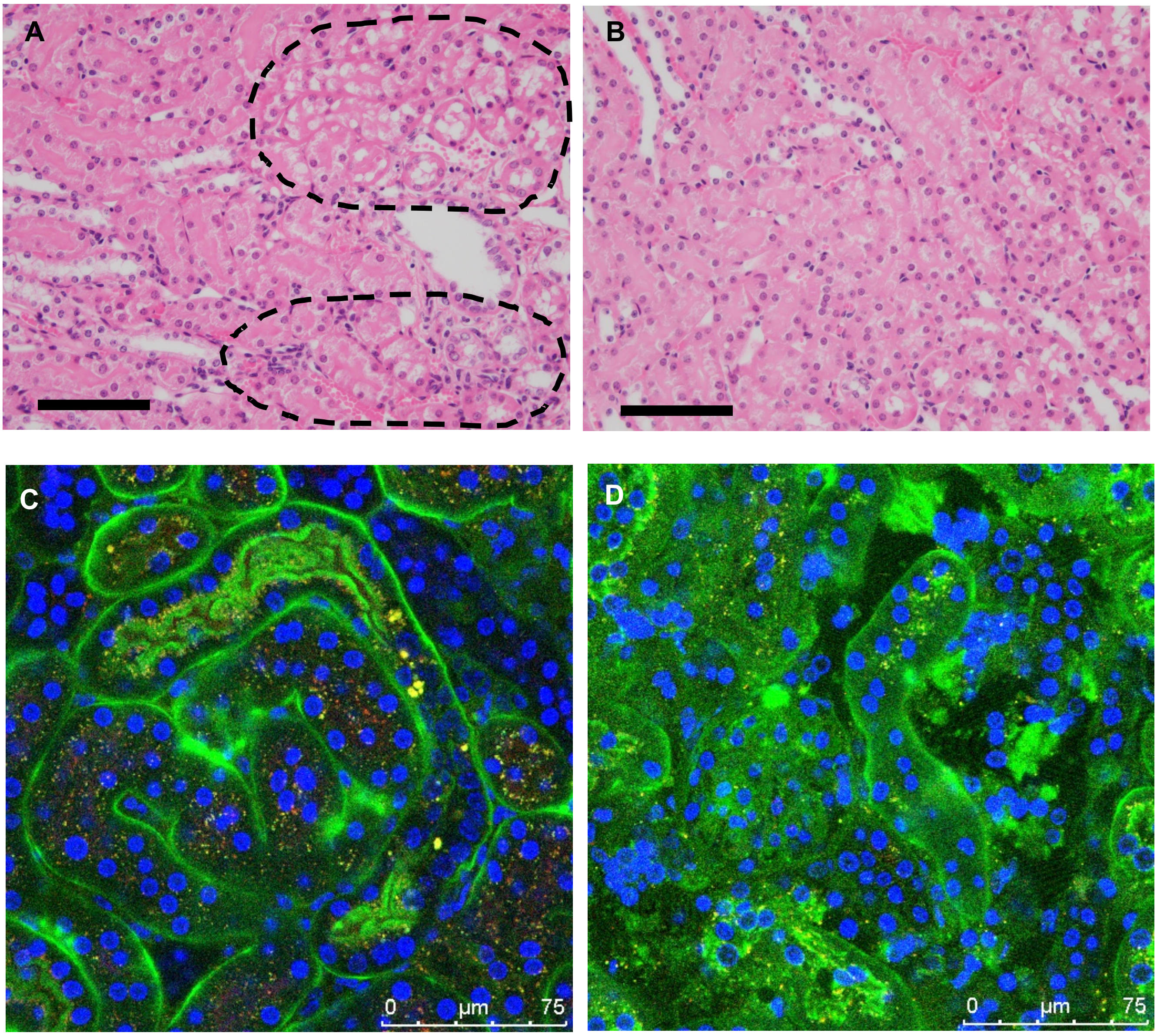

Supplementary Figure S5 | Effects of the triple dose sRANKL on renal histology. Histological analysis of the kidneys from the mice treated with sRANKL or vehicle in the triple dose regimen at 36 hours after the last injection. Representative images of hematoxylin-eosin staining from an sRANKL-treated mouse (**A**) and a vehicle-treated mouse (**B**). Bar = 100 μm. The sRANKL-treated mice exhibited tubulointerstitial damage, including vacuolar degeneration and interstitial cell infiltration (areas enclosed by dotted line). Representative ex vivo CPP images of the kidneys from an sRANKL-treated mouse (**C**) and a vehicle-treated mouse (**D**). The interstitial space and the apical membrane of proximal tubules are visualized with FITC (green). Cell nuclei are stained with Hoechst (blue). CPP containing calcium phosphate crystals were detected as small red spots or yellow spots when overlaid with FITC in the tubular lumen (**C**).

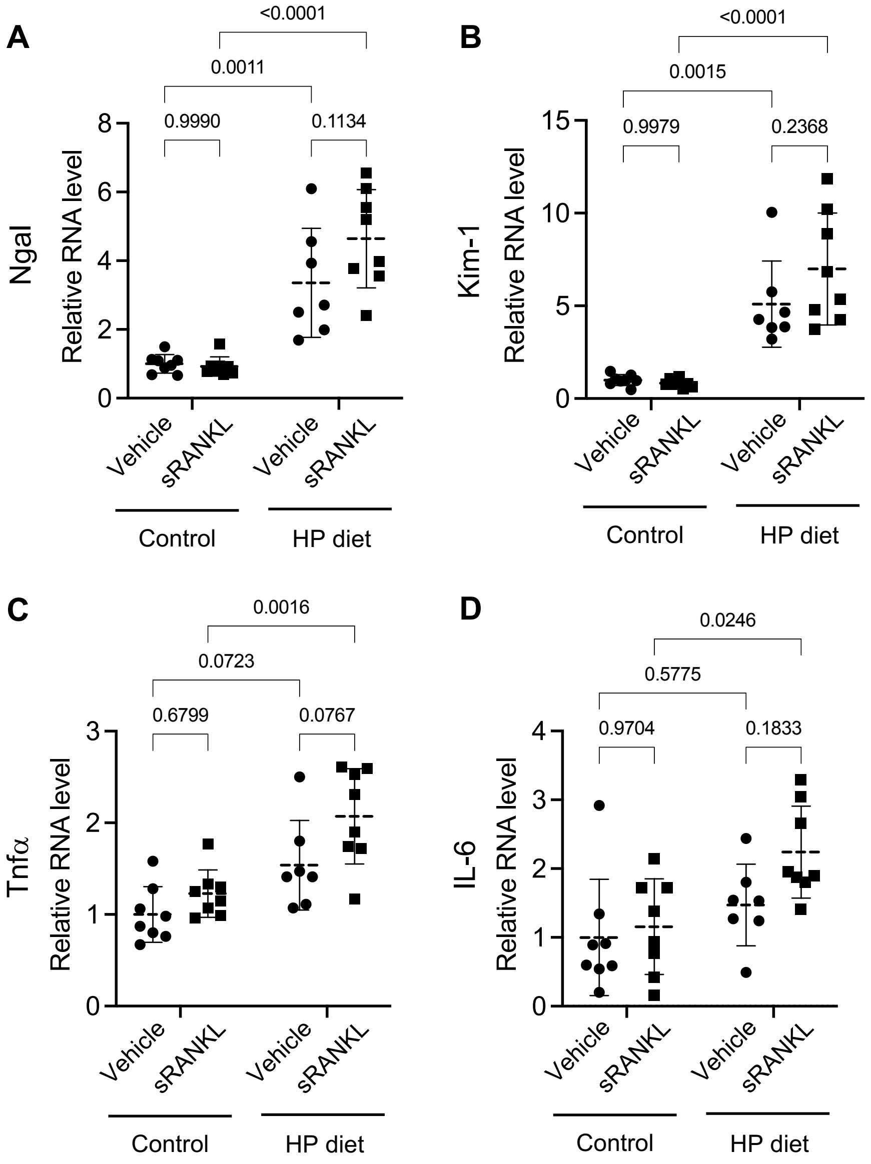

Supplementary Figure S6 | Effects of sRANKL treatment on expression levels of kidney injury markers in mice with or without dietary phosphate loading. Mice were fed either a regular diet containing 0.35% inorganic phosphate (Control) or a high-phosphate diet containing 1.5% inorganic phosphate (HP diet). Relative mRNA levels of Ngal (A), Kim-1 (B), Tnfα (C), and IL-6 (D). Data are presented as means ± SD. N = 7~8 per group. Statistical significance was assessed by two-way ANOVA followed by Tukey's multiple-comparison test; P values are indicated.

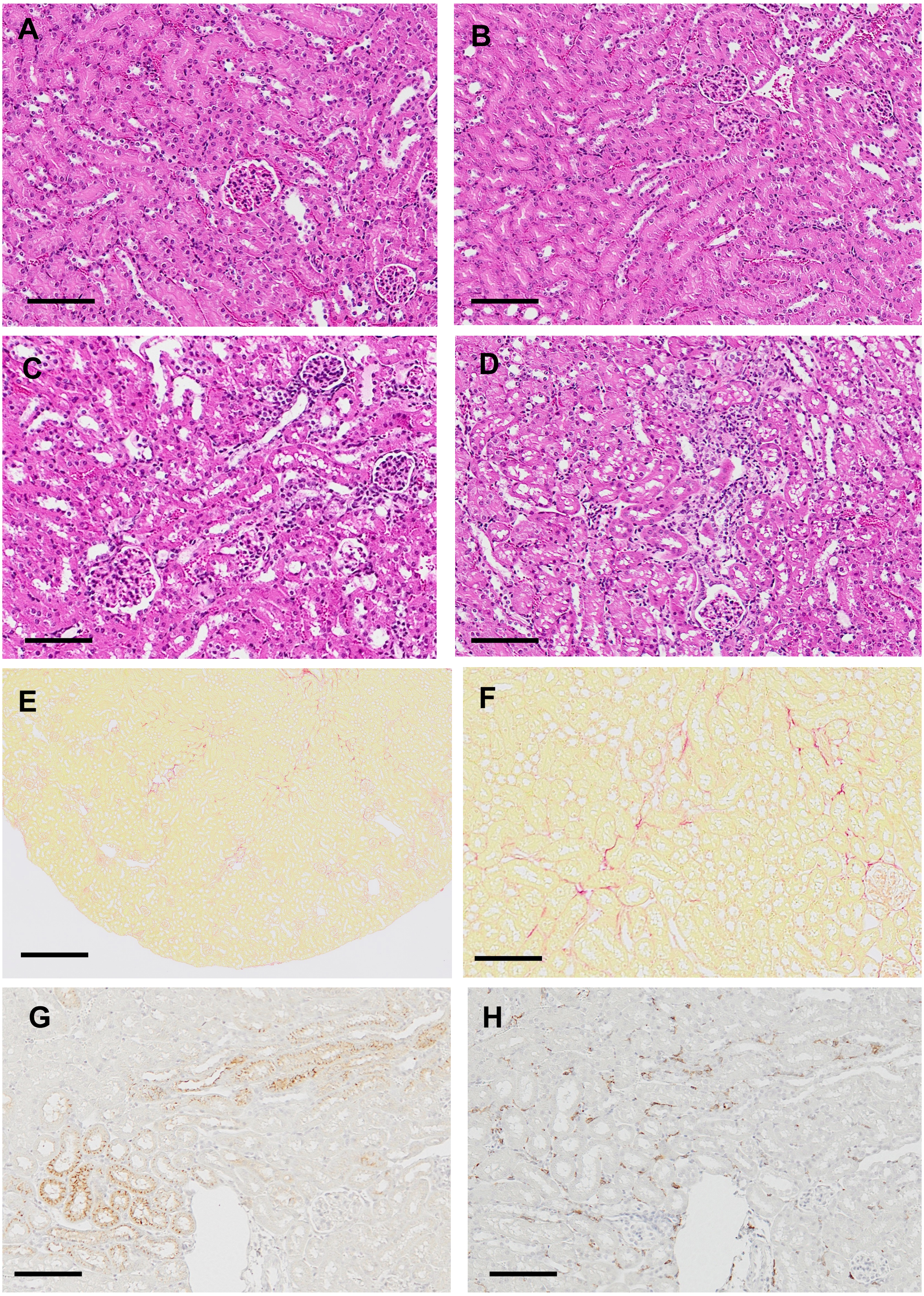

Supplementary Figure S7 | Effects of sRANKL treatment on renal histology in mice with or without dietary phosphate loading. Hematoxylin-eosin staining revealed no tubular damage or inflammatory cell infiltration in control mice treated with vehicle (**A**) or sRANKL (**B**). In mice fed a high phosphate diet, tubular vacuolization and inflammatory cell infiltration were evident with both vehicle (**C**) and sRANKL treatment (**D**). Sirius Red staining demonstrated interstitial fibrosis in a mouse fed the high phosphate diet treated with sRANKL (**E**, low magnification; **F**, high magnification). Immunohistochemistry for osteopontin (**G**) and F4/80 (**H**) in a CKD mouse treated with sRANKL. Representative images from six mice in each group are shown. Scale bars: 100 μm (**A-D**), 400 μm (**E**), 200 μm (**F-H**).

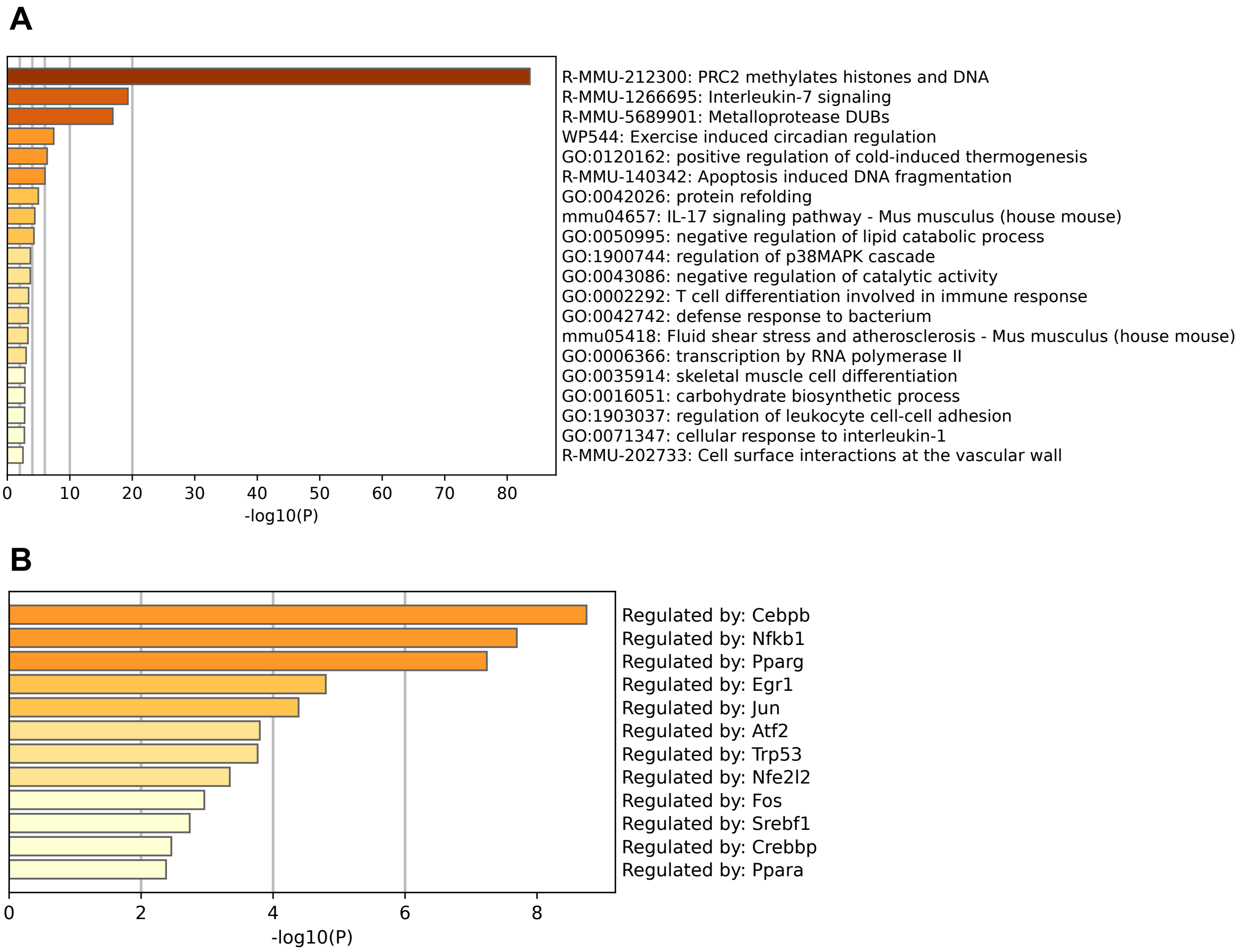

Supplementary Figure S8 | Pathways and transcriptional regulatory networks up-regulated under microgravity in bones. Genes up-regulated >1.5-fold in microgravity were subjected to enrichment analysis. Significantly enriched terms (**A**) and TRRUST (transcriptional regulatory relationships unravelled by sentence-based text-mining) (**B**) are shown according to p-values.

A

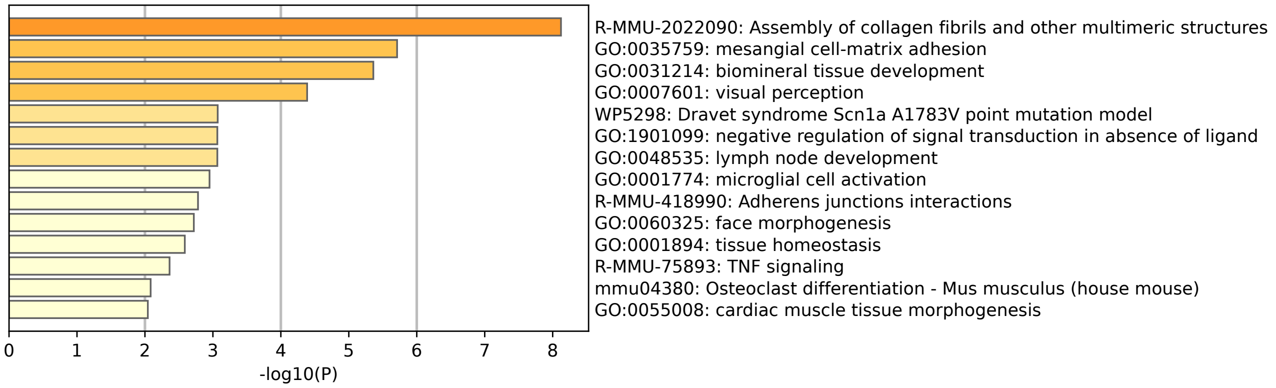

B

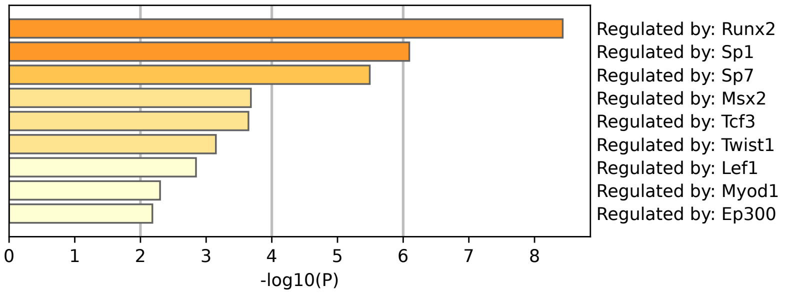

Supplementary Figure S9 | Pathways and transcriptional regulatory networks down-regulated under microgravity in bones. Genes down-regulated < 1/1.5-fold in microgravity were subjected to enrichment analysis. Significantly enriched terms (**A**) and TRRUST (transcriptional regulatory relationships unravelled by sentence-based text-mining) (**B**) are shown according to p-values.

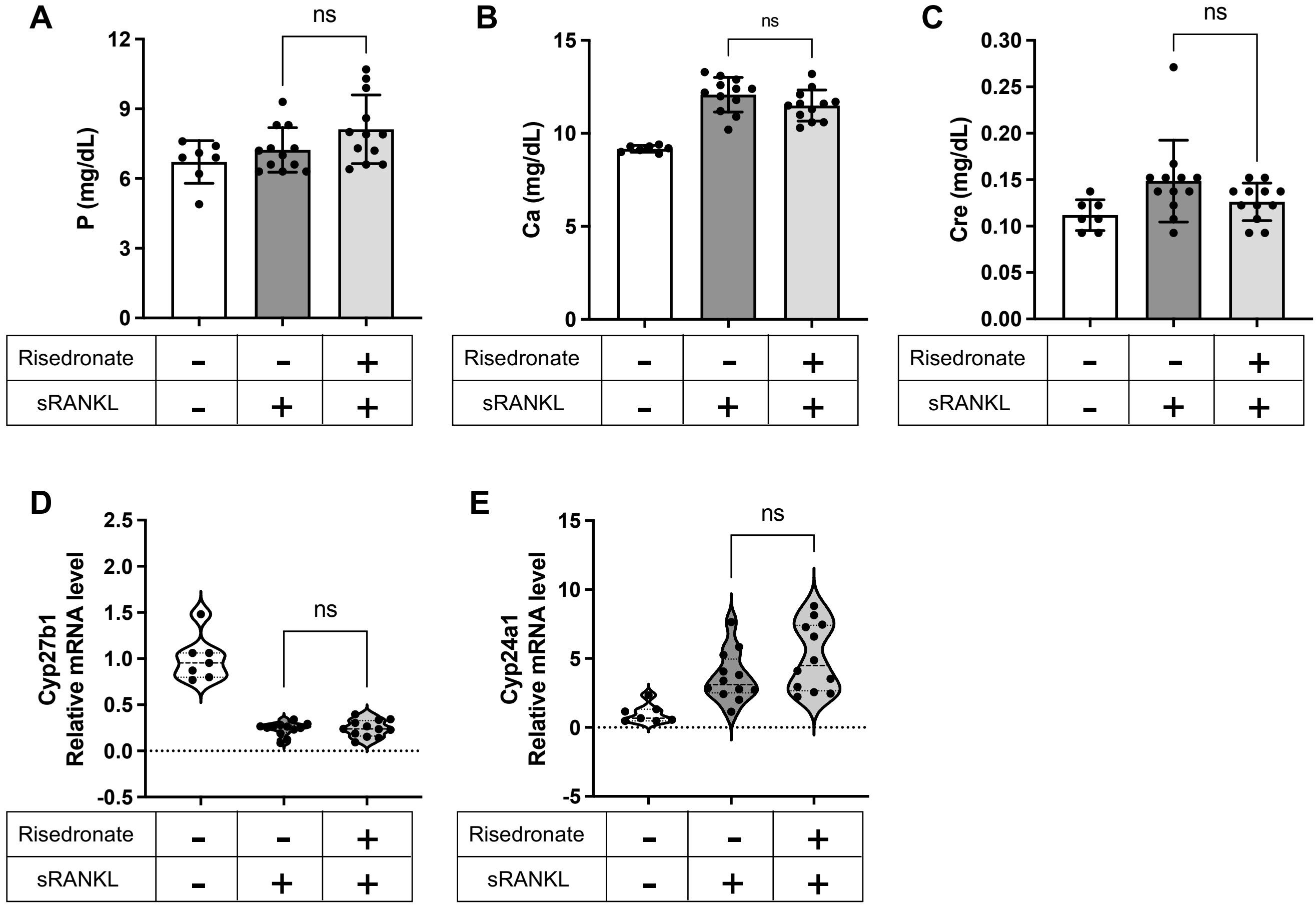

Supplementary Figure S10 | Effects of risedronate on mice treated with the triple dose regimen of sRANKL. Plasma and kidney samples from **Figure 4** were further analyzed. Plasma levels of phosphate (**A**), calcium (**B**), and creatinine (**C**). Data are presented as means ± SD. N = 6-12 for each column. The risedronate treatment did not affect these plasma parameters in mice treated with the triple dose sRANKL as determined by one-way ANOVA with Tukey's multiple comparison test (ns). Relative mRNA levels of *Cyp27b1* (**D**) and *Cyp24a1* (**E**) in the kidney were measured by quantitative RT-PCR. Data are indicated as violin plots with the median and quartiles (dotted lines). N = 6 for each column. The risedronate treatment did not affect expression of these genes in mice treated with the triple dose sRANKL as determined by Kruskal–Wallis's test with Dunn's multiple-comparison test (ns).

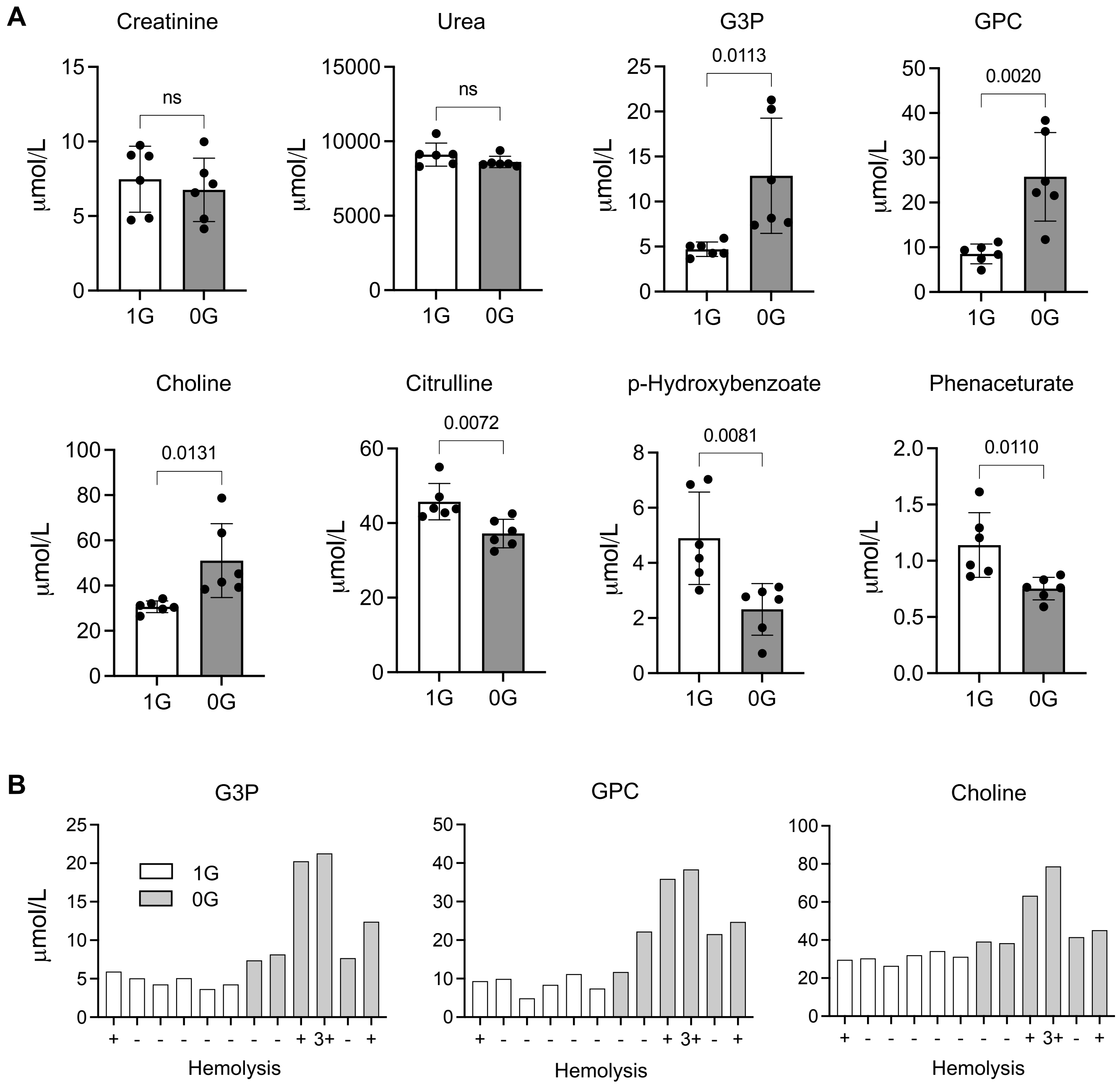

Supplementary Figure S11 | Plasma metabolomics in spaceflight mice and ground control mice. (**A**) Hydrophilic metabolites significantly different between the spaceflight mice (0G) and the ground control mice (1G). G3P, glycerophosphate (glycerol-3-phosphate); GPC, Glycerophosphorylcholine. Data are means ± SD; n = 6 for each column. *P* values by t-test are indicated. (**B**) Effects of hemolysis on plasma G3P, GPC, and choline concentrations. The degree of hemolysis was indicated in individual samples (-, none; +, mild; 3+, marked).
