# Supplementary material for: Bone mineral loss damages renal tubules in mice
Source: Commun Biol. 2026 Jan 22;9:304. doi: 10.1038/s42003-026-09603-0 (PMC12929778; doi:10.1038/s42003-026-09603-0)
Supplement: Supplementary file 3 — Description of Additional Supplementary Files [file 42003_2026_9603_MOESM3_ESM.pdf]

**Description of Additional Supplementary File**

File Name: Supplementary Data 1

Description: Numerical Source Data

File Name: Supplementary Data 2

Description: Spaceflight Mice Bone RNAseq

File Name: Supplementary Data 3

Description: Spaceflight Mice Plasma Metabolome
